# Octave-spanning coherent supercontinuum generation in silicon on insulator from 1.06 μm to beyond 2.4 μm

**DOI:** 10.1038/lsa.2017.131

**Published:** 2018-01-26

**Authors:** Neetesh Singh, Ming Xin, Diedrik Vermeulen, Katia Shtyrkova, Nanxi Li, Patrick T Callahan, Emir Salih Magden, Alfonso Ruocco, Nicholas Fahrenkopf, Christopher Baiocco, Bill P-P Kuo, Stojan Radic, Erich Ippen, Franz X Kärtner, Michael R Watts

**Affiliations:** 1 Research Laboratory of Electronics, Massachusetts Institute of Technology, Cambridge, MA 02139, USA; 2 John A. Paulson School of Engineering and Applied Science, Harvard University, Cambridge, MA 02138, USA; 3 College of Nanoscale Science and Engineering, SUNY Polytechnic Institute, Albany, NY 12203, USA; 4 Department of Electrical and Computer Engineering, University of California San Diego, La Jolla, CA 92039, USA; 5 Centre for Free Electron Laser Science (CFEL)-DESY and University of Hamburg, Hamburg 22607, Germany

**Keywords:** coherence, integrated photonics, silicon, supercontinuum

## Abstract

Efficient complementary metal-oxide semiconductor-based nonlinear optical devices in the near-infrared are in strong demand. Due to two-photon absorption in silicon, however, much nonlinear research is shifting towards unconventional photonics platforms. In this work, we demonstrate the generation of an octave-spanning coherent supercontinuum in a silicon waveguide covering the spectral region from the near- to shortwave-infrared. With input pulses of 18 pJ in energy, the generated signal spans the wavelength range from the edge of the silicon transmission window, approximately 1.06 to beyond 2.4 μm, with a −20 dB bandwidth covering 1.124–2.4 μm. An octave-spanning supercontinuum was also observed at the energy levels as low as 4 pJ (−35 dB bandwidth). We also measured the coherence over an octave, obtaining 
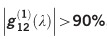
, in good agreement with the simulations. In addition, we demonstrate optimization of the third-order dispersion of the waveguide to strengthen the dispersive wave and discuss the advantage of having a soliton at the long wavelength edge of an octave-spanning signal for nonlinear applications. This research paves the way for applications, such as chip-scale precision spectroscopy, optical coherence tomography, optical frequency metrology, frequency synthesis and wide-band wavelength division multiplexing in the telecom window.

## Introduction

The ultimate legacy of nonlinear optics is considered by many to be the extreme spectral broadening of light in supercontinuum generation (SCG), which comprises many of the most important nonlinear phenomena. This effect has come a long way from its discovery in bulk media by Alfano and Shapiro in 1970^1^ to the advent of photonic crystal fibers for efficient SCG by providing control over the mode area and therefore the group velocity dispersion (GVD)^2^. The high coherence and brightness of a supercontinuum (SC) enable applications in frequency metrology and synthesis^3^, spectroscopy^4^, optical coherence tomography^5^ and ultra-short tunable pulses^6^, and as a broad-band source for wavelength division multiplexing^7^.

Nevertheless, to become mainstream in nano-photonics, SC devices need to leverage complementary metal-oxide semiconductor (CMOS)-compatible materials, such as silicon. Silicon in its common form of silicon on insulator (SOI) has been used extensively in linear photonics for providing compact architectures because of the high index contrast between the silicon core and the silica cladding^8, 9^. Silicon has a high Kerr coefficient (*n*_2_), 3, 30 and 400 times higher than that of chalcogenide, silicon nitride and silica, respectively, in the telecom window^10^, facilitating power-efficient nonlinear devices^11^. However, nonlinear loss such as two-photon absorption (TPA) has impeded the realization of the potential of the use of silicon as a nonlinear platform in the telecom window^12, 13, 14^. TPA is a process in which an electron in the valence band absorbs two incoming photons simultaneously to be excited above the bandgap and contributes to the loss of input signal. This loss is exacerbated by continued free carrier absorption.

To avoid TPA in silicon, nonlinear photonics research in the near-IR is slowly drifting toward non-silicon platforms such as silicon nitride^15^, Hydex^16^, chalcogenide^17^, silicon-germanium^18^, indium gallium phosphide^19^ and aluminum nitride^20^. Alternatively, a modified version of silicon, hydrogenated amorphous silicon (a-Si:H), has also been introduced that essentially increases the bandgap of the material from 1.12 to 1.6 eV^21^. This increase in bandgap causes the TPA edge to be pushed closer to the visible, increasing the nonlinear figure of merit, which is the ratio of the Kerr coefficient to TPA. However, no octave-spanning SC has been observed to date, except in the exotic amorphous silicon fiber structure in the mid-infrared (mid-IR)^22^.

The obstacle posed by TPA has indeed pushed octave-spanning SC research into the long wavelength region^23, 24, 25, 26, 27^. For example, octave-spanning SC in SOI has been demonstrated in the shortwave infrared (SWIR)^23^ (−30 dB bandwidth), albeit with absorption losses beyond 2.5 μm in the presence of the silica substrate. To circumvent silica loss, a mid-IR transparent silicon on sapphire waveguide^28, 29^ has been shown to have a SC spanning from 1.9 to 6 μm^26^ (−20 dB octave), with the SC predicted to reach up to 8 μm^30, 31^. In addition to operating in the mid-infrared, these devices require integrated mid-IR mode-locked lasers for full integration; this challenge has not been addressed yet.

Nevertheless, there are recent theoretical studies of octave-spanning SC in the near-IR (spanning 1.2–2.4 μm) using 60 fs pump pulses at 1.8 μm in a horizontal slot waveguide manifesting four zero dispersion wavelengths (ZDWs)^32^. However, there are no reports yet of the experimental demonstration of SCG in these structures. Furthermore, for applications such as on-chip frequency metrology, it would be required to demonstrate that the octave-spanning SC maintains the spectral coherence of the input pulses. Recent work on the coherence of the SC in SOI waveguides has demonstrated coherence for just over half an octave in the near-IR^33^, and part of the SC when measured in the SWIR^23^.

In this work, we experimentally demonstrate octave-spanning SC generation in SOI ridge waveguides from the edge of the silicon transmission window, ~1.06 μm, up to the SWIR (beyond 2.4 μm) with −20 dB bandwidth covering 1.124–2.4 μm range. A high degree of coherence (>90%) is measured over the full octave, in good agreement with simulations. We also experimentally show the role of third-order dispersion for efficiently generating dispersive waves. Furthermore, advantages of a soliton for nonlinear processes at the long wavelength edge of an octave-spanning signal over the dispersive waves beyond 2nd ZDW are highlighted.

This work, along with the recent achievement of efficient second-harmonic generation in silicon^34^ and integrated mode-locked laser^35^, will have immediate applications in silicon-based on-chip optical atomic clock and frequency synthesis.

## Materials and methods

### Waveguide design

**Figure 1:**
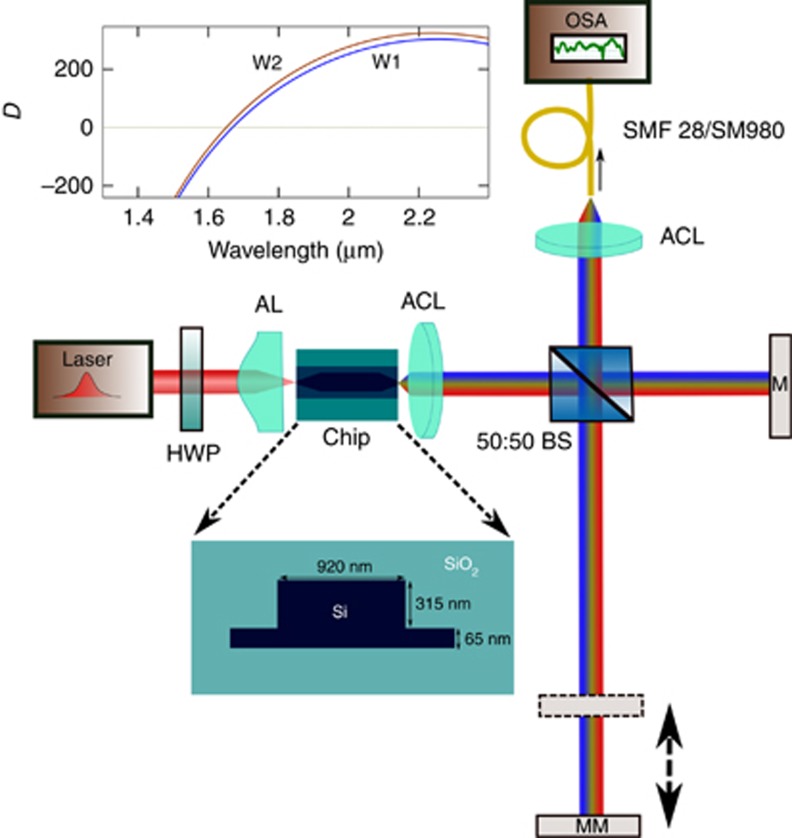
The asymmetric Michelson interferometer setup for measuring the SC and its coherence, 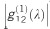. The SC is measured with a multimode InF_3_ fiber butt coupled at the output (not shown). ACL, achromatic lens; AL, aspheric lens; BS, beam splitter; HWP, half wave plate; MM, movable mirror. SMF28/SM980 are single-mode fibers to measure coherence above 1.4 μm and below 1.4 μm, respectively. The cross-section of the SOI waveguide (W1) is shown with the dispersion of W1 and W2 waveguides in the inset. Unit of *D* is ps nm^−1^ km^−1^.

To achieve a broad SC bandwidth reaching close to the silicon edge, we designed two waveguides to be pumped at slightly different wavelengths around 1.9 μm. We chose 1.9 μm because the intensity-dependent refractive index (*n*_2_) of silicon is two times higher and TPA is three times lower than that at 1550 nm^36^. The SC waveguide (W1) was designed for optimal dispersion for the fundamental quasi-TE mode at 1.9 μm. As shown in Figure 1, the width, height and the slab thickness are 920, 315 and 65 nm, respectively. The dispersion profile shown in Figure 1 (inset) exhibits optimum higher-order dispersion (greater than GVD), to generate the signal (dispersive wave) close to the silicon edge (≈1.1 μm). The 1st ZDW is ~1.67 μm and the GVD at the pump wavelength is approximately 0.4 ps^2^ m^−1^. To further improve and reach below 1.1 μm, we designed another waveguide (W2) with slightly different width (0.9 μm) to be pumped at 1.95 μm, having dispersion as shown in Figure 1 (inset). Even though the waveguides are supporting a higher-order mode, due to a markedly different dispersion profiles of the different modes, the intermodal coupling and the resulting nonlinear wave mixing are expected to be negligible^13, 37^. To improve input coupling, the waveguides are integrated with inverse horizontal tapers^38^. The taper lengths were 130 μm, linearly increasing from the edge of the chip from the tip width of 160 nm to the waveguide width. The short taper length was chosen to minimize the contribution from the taper to the nonlinear effects. We note that further improvements in the taper design are required for efficient free space coupling.

The waveguides were fabricated within a 300 mm line in a standard CMOS facility by epitaxially growing silicon to the thickness of 380 nm on a 220 nm SOI wafer. The wafer was then exposed and patterned using 193 nm deep-ultraviolet immersion lithography and subsequently partially etched down to 315 nm using reactive-ion etching to obtain the ridge waveguide.

## Results and discussion

### Supercontinuum measurements

The source was a 200 MHz, 1550 nm fiber laser producing ~100 fs pulses, which were Raman shifted in a highly nonlinear fiber, producing pulses of >50 fs duration at 1.9 μm (Menlo Systems—HMP:EDFA). The estimated on-chip power in the waveguide was ~3.6 mW (18 pJ). As shown in Figure 1, the light was coupled using an aspheric lens (Black diamond, NA-0.56 with 95% transmission at 1.9 μm). The coupling to the chip was ensured by two techniques: first, we used an IR camera to observe a faint streak of scattered light along the surface of the waveguide and then observed (by naked eye/CCD camera) a red-light spot at the input facet of the waveguide due to 3rd harmonic conversion^39^. The output was collected using a SWIR transparent InF_3_ multimode fiber (100 μm core) butt coupled to the waveguide and measured using two optical spectrum analyzers (OSA's) from Yokogawa-AQ6370D (up to 1700 nm) and AQ6375B (up to 2400 nm).

**Figure 2:**
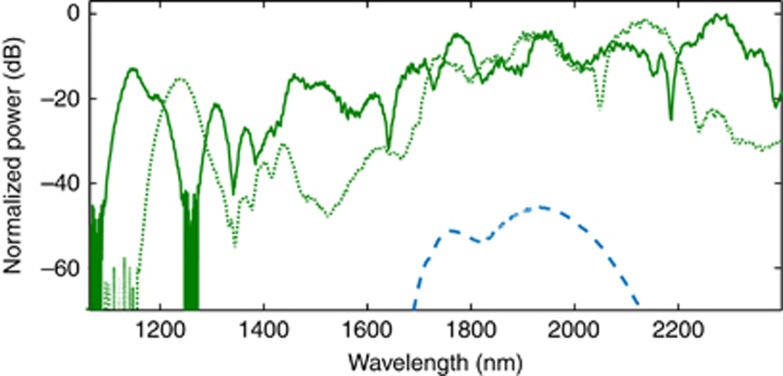
The SC spectrum (in green) with SOI waveguide W1. The pump spectrum (blue dashed line) is centered at ~1.9 μm. The SCG extends from 1.1 μm to over 2.4 μm (18 pJ coupled energy). The dotted signal was measured with coupled energy of 4 pJ. Note that the noise level difference between the solid and dotted curves is due to the change in the sensitivity level of the spectrum analyzer.

**Figure 3:**
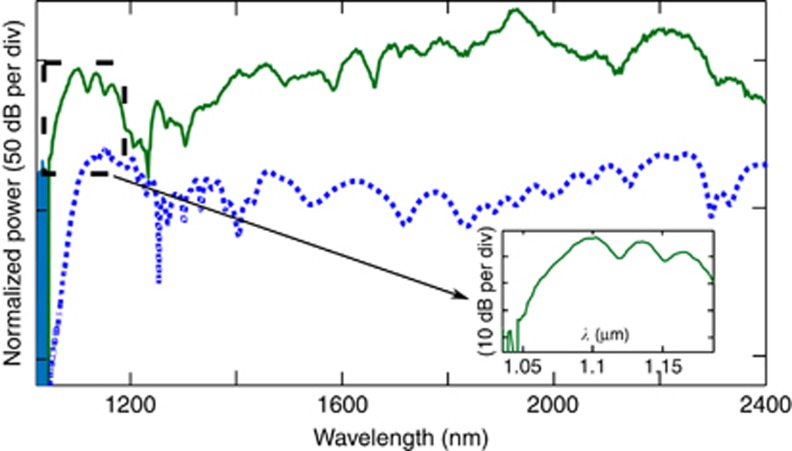
The SC spectrum with a waveguide (W2) dimension width of 0.9 μm, height of 0.315 μm and slab thickness of 0.065 μm. The SC (in green) extends from 1.06 μm to over 2.4 μm when pumped at 1.95 μm. The simulation is shown in blue dots. The inset shows the magnified image of the signal at the short wavelength side of the SC showing signal reaching below 1.1 μm.

The measured supercontinuum is shown in Figure 2. The −20 dB SC ranges from 1.124 to 2.40 μm with strong signals at 1*f* (2.30 μm) and *2f* (1.15 μm). The power spectral density on chip at *f* was expected to be greater than −25 dBm nm^−1^ and at *2f*>−37 dBm nm^−1^. We also measured octave-spanning SC with coupled energy as low as 4 pJ (shown in dotted green in Figure 2) at a −35 dB bandwidth. We note here that improvement in the overall signal strength is expected by using a transformed limited pump source. The waveguide W2 was pumped with similar laser pulse parameters except that the center wavelength was ~1950 nm. As shown in Figure 3, SC was observed from 1.06 μm extending beyond 2.40 μm, with a 45 nm signal improvement at the short wavelength side compared to the W1 waveguide. This result is the shortest wavelength extension observed in silicon SC so far. The *f* and *2f* peaks are shifted to 1.1 and 2.2 μm compared with those of the W1 waveguide. For both waveguides discussed here, the propagation loss was measured to be 1.5 dB cm^−1^ around the pump wavelength.

### Coherence measurements

For the applications discussed in the Introduction, it is important to ensure that the SC process preserves the spectral coherence of the mode-locked laser, that is, avoids the degradation in the phase correlation between the comb lines. In silicon SC, the degradation normally occurs due to the amplification of the noise present in the pulse by the modulation instability (MI)^40^. MI occurs mainly when operating in the anomalous GVD region and can be interpreted as a four-wave mixing process seeded by noise^41^. MI usually plagues SCG with longer pulses and longer waveguides because of sufficient time and length for MI development. By using shorter pulses (sub 100 fs) and a waveguide (slightly longer than soliton fission length), coherence degradation due to MI can be alleviated^27^.

**Figure 4:**
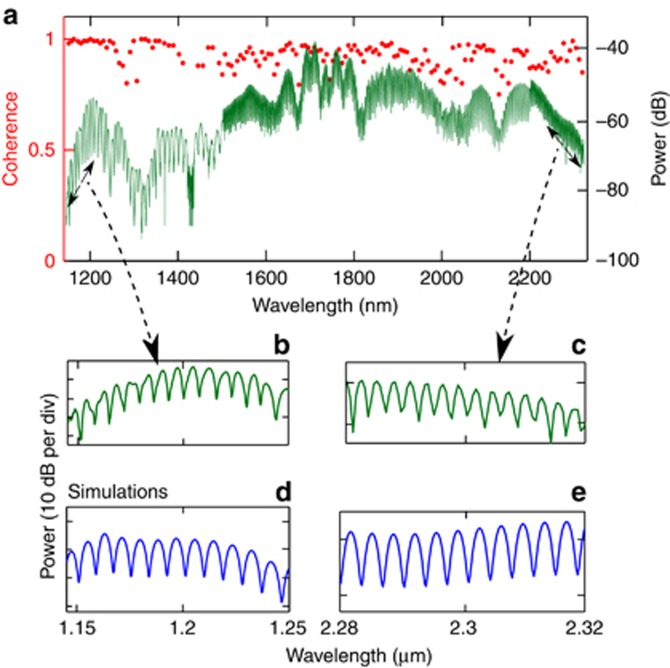
Measured coherence and visibility fringes of the SC (W1). (**a**) Octave-spanning visibility fringes (right axis). The signal span from 1.15 to 2.2 μm is taken with 4 dB loss, and that from 2.2 to 2.32 μm is taken with 6 dB loss in the long arm. (**b**,**c**) Magnified range of SC fringes with periods 8 and 3 nm, respectively. Because of the differences in delay, the period of fringes varies, but the fringe visibility is not affected. Panels (**d**) and (**e**) are the simulated fringes.

To quantify the coherence of non-stationary light^40, 42^ such as SC pulses, we need to determine the modulus of the complex degree of first-order coherence, given as follows:


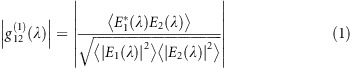


which is an ensemble average of many pairs of SC spectra. Here, *E*_1_ and *E*_2_ represent electric fields of individual SC. The coherence can be experimentally measured by observing an interference pattern using the setup shown in Figure 1^43, 44, 45^, which is an asymmetric Michelson interferometer. The asymmetric arm is required to overlap two different SC pulses in time, which are then sent to the OSA, whereas the movable mirror controls the delay d*t* between the pulses. The coherence is thus represented as follows^44^:


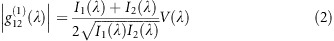


where *I*_1_ and *I*_2_ are the intensities of the beams in the two arms, and *V* is the fringe visibility (at each wavelength) obtained as *V*=(*I*_max_*−I*_min_)/(*I*_max_+*I*_min_). The SC spectrum with the interference fringes (W1) is shown in Figure 4a.

The signal was only observed in the 1.146–2.32 μm range due to the divergence of the beams caused by the limited bandwidth of the achromatic lenses. By changing d*t*, the fringe period could be varied without affecting the visibility. From 1.15 to 1.40 μm, the period is ~8 nm (d*t*≈0.55 ps), whereas at ~2.3 μm, the period is close to 3 nm (d*t*≈5 ps). We also had a weaker signal in the longer arm than in the shorter one due to the beam divergence discussed above. The intensity difference between the two arms (*I*_1_−*I*_2_) for the spectrum above and below 2.2 μm was approximately 6 and 4 dB, respectively. This result caused the difference in contrast seen in Figure 4b and 4c. Intuitively, the observance of fringes can be understood by decomposing a pulse train into its constituent CW modes. Each mode creates a fringe pattern (as in a conventional Michelson interferometer) that overlaps with the fringes of the other modes in space. When a detector is placed in a region along these overlapping fringes, a frequency-dependent pattern is observed, as shown in Figure 4. With the losses taken into account, the coherence is plotted in Figure 4a (red dots). We see that the coherence is >90% on average in the measured spectrum. Moreover, in the *f* and 2*f* regions, the coherence is higher than 95% and 92%, respectively (over 30 nm bandwidth).

### Numerical simulation of the supercontinuum

**Figure 5:**
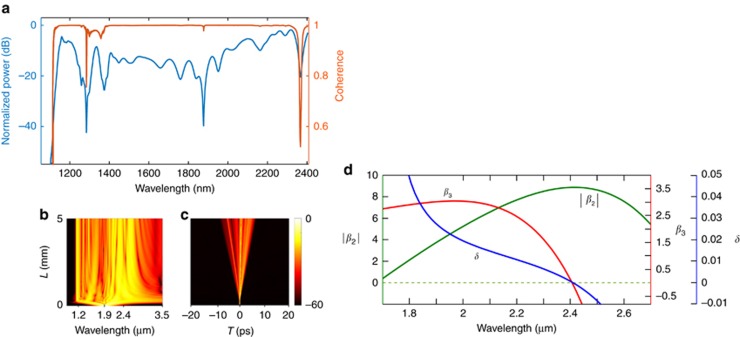
(**a**) Simulation of the SCG and coherence, 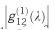 produced by waveguide (W1). (**b**,**c**) Spectral and temporal evolution of the SC along the waveguide. (**d**) Simulated third-order dispersion *β*_3_ × 10^−39^ s^3^ m^−1^ (in red), modulus of group velocity dispersion *β*_2_ × 10^−25^ s^2^ m^−1^ (in green) and the unitless third-order dispersion parameter *δ* (in blue).

The SCG and its coherence were simulated by numerically solving a generalized nonlinear Schrodinger equation (NLSE) using an adaptive split step^41^. The simulated SC for the waveguide (width of 0.92 μm) is shown in Figure 5a. We used 50 fs transform limited sech pulses with a repetition rate and energy of 200 MHz and 18 pJ, respectively. The peak ~1.2 μm (near *2f*) is mainly due to the dispersive wave (DW) generation^46^. The DW can be selectively tuned by realizing that they are generated mainly by the perturbation of the input pulse by the third-order dispersion because the self-steepening and Raman effects are negligible in our waveguides^41, 47^. The amplitude and wavelength shift of DW can be approximated by the third-order dispersion effect given as follows^48^:


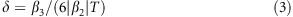


where, *β*_3_, *β*_2_ and *T* are the third- and second-order dispersions and the temporal width of the pulse, respectively. The *δ*, *β*_3_ and the modulus of *β*_2_ are plotted for W1 in Figure 5d. The *δ* represent the strength of the third-order perturbation on the input pulse^47, 48^. Generally, for a given power, the wavelength shift of a dispersive wave decreases with increasing *δ* while its amplitude increases^48^. This occurs because higher *δ* corresponds to the pump wavelength being closer to the ZDW; hence, its phase matches mainly to the DWs closer to the ZDW. However, with higher *δ*, the third-order perturbation on the pump pulse is also higher; thus, it releases more radiation into the DW. By contrast, a smaller *δ* increases the *2f* shift; however, as the *β*_3_ becomes too low, the higher-order dispersion effects can produce dispersive waves beyond 2nd ZDW^49^, reducing the 2*f* shift. Therefore, there is a trade-off between the achievable DW amplitude and the frequency shift, which was considered in the design of our waveguide for pumping at 1.9 μm wavelength. The *δ* at 1.9 μm is ~0.022 for W1, generating strong DW at 2*f* and around the telecom window (more details in the next section). We must note that by reducing the slab thickness of the waveguides, *δ* can be reduced further, resulting in a shorter *2f* (given no band-edge loss by silicon) while shifting the *f* signal closer to the pump as GVD increases.

Following the soliton fission of the pump pulse around 0.3 mm, the DWs start building up in the normal dispersion region as shown in Figure 5b and 5c). The fission length is given as ≈*L*_d_/*N*, where *L*_d_ is the dispersion length (*L*_d_=*T*_*o*_^2^/*β*_2_=6.4 mm, *T*_*o*_ is the pulse width, *β*_2_ is GVD at 1.9 μm) and *N* (=18) is the soliton number given as, 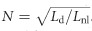. Here, *L*_nl_ is the nonlinear length equal to 1/*γP*, where *γ*=142 mW^−1^ and *P* is 360 W. Waves leading the center pulse are observed in Figure 5c, where they are generated in the region where the silica substrate exhibits strong absorption. The slight discrepancy between the spectra could be due to the fabrication tolerance in the dimensions of the waveguide and the initial chirp of the pump pulse. The waveguide W2 was also simulated and the results are shown in Figure 3 (blue dots). The estimated soliton number is 18 for W2. We observe that the dispersive wave (in the *2f* region) is slightly shorter than what is measured. This discrepancy could be mainly due to the sensitivity of dispersion to the fabrication tolerances^26^.

The coherence was calculated using Equation (1) for which 100 SC pairs were calculated. Each SC pulse incorporated one photon per mode fluctuation and 1.5% of intensity noise^50^. The result is shown in Figure 5a and matches well with the experimental data. We also simulated the fringes at the *f* and 2*f* spectral regions with the delay between the pulses approximately 5 and 0.55 ps, respectively, as shown in Figure 4d and 4e. The fringes match well with the experimental results, even though there is a slight discrepancy between Figure 4c and 4e that could be due to the uncertainty in the delay and/or loss in each arm. The coherence can be further improved by reducing the soliton fission length (by reducing the dispersion length, *L*_d_) and the physical length of the waveguide, before significant amplification of input noise by MI can occur^40^.

### Selective signal improvement

**Figure 6:**
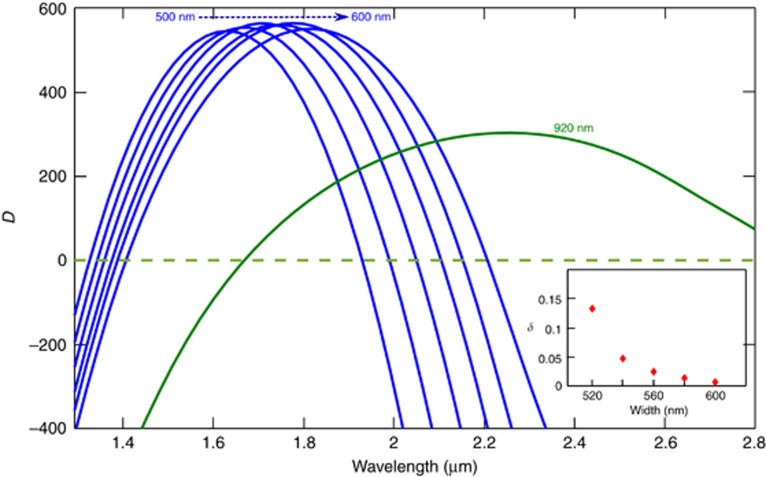
Dispersion of waveguide varying in width from 500 to 600 nm (in blue) shown along with W1 (green) for comparison. The inset shows *δ* for waveguides at 1.94 μm. Unit of *D* is ps nm^−1^ km^−1^.

Specific spectral windows in the supercontinuum can be selectively improved; for example, in this work we tried increasing the signal strength around 1*f* (2.3 μm), 2*f* (1.15 μm) and 1550 nm with a straight waveguide. For 1*f*, we utilized solitons as opposed to long dispersive waves (LDWs) that are generated beyond 2nd ZDW and normally require a waveguide with two spectrally neighboring ZDWs^25, 51, 52, 53^. This strategy was motivated by the following considerations: (a) solitons are shorter pulses with higher peak power than the LDWs, and (b) the temporal delay between short dispersive wave (SDW) (*2f*) and soliton (*f*) is relatively lower compared to SDW and LDW (*f*) combination, which will be useful for applications in which further nonlinear conversion is required, such as the SHG process in the self-referencing applications. To test the strength of the soliton at *f* compared to LDW, we designed and tested several waveguides with varying widths with 20 nm increments from 500 to 600 nm (height and slab thickness same as W1), with 2nd ZDW close to the pump wavelength (~1.94 μm) as shown in Figure 6. The 2nd ZDW ranges from 1.93 to 2.2 μm for 500–600 nm wide waveguides, respectively.

**Figure 7:**
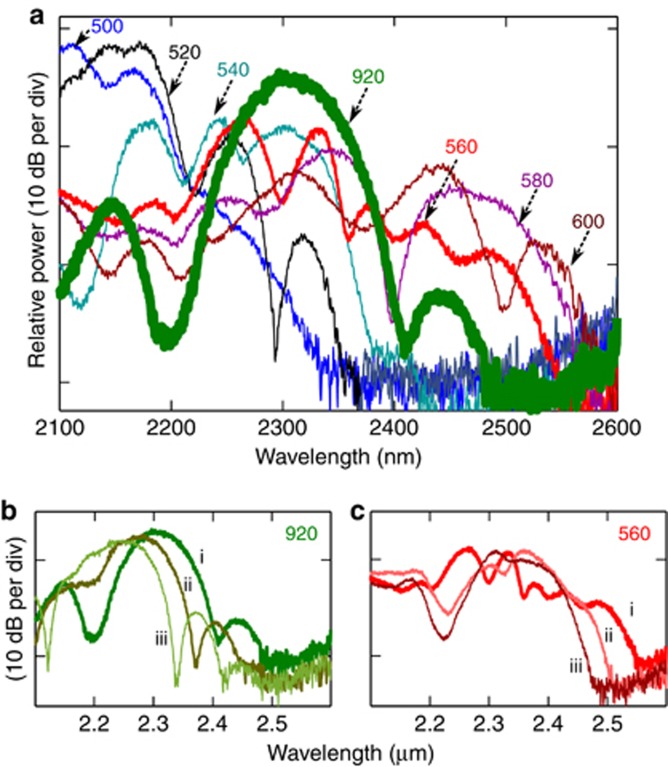
(**a**) Measured long-wave dispersive wave (LDW) from waveguides 500–600 nm in width. The soliton signal at 2.3 μm is shown from W1 (in green). (**b**,**c**) pump power response of the soliton signal at 2.3 μm of W1 and LDW of 560-nm-wide waveguide, respectively. Here, i, ii and iii represent 30, 21 and 10 pJ of coupled energy. All the waveguides were pumped at 1.94 μm with same input power.

All waveguides were pumped with the same source discussed above, with the center wavelength shifted to 1.94 μm at the energy of 30 pJ. The results are shown in Figure 7a. The input coupling for each waveguide was optimized until the LDW was generated to the longest possible wavelength, which is a sign of maximum coupling. Here we observe that the wider the waveguide the further the LDW shift. We also tested waveguides with widths >600 nm but the overall signal strength kept decreasing, especially in the region of interest (*f*~2.3 μm). The observed spectra can be explained based on the *δ* parameter as discussed in the previous section. We calculated the *δ* for all waveguides (shown in the inset of Figure 6), and it can be seen that *δ* values decrease with increasing waveguide width. We did not plot *δ* for the 500-nm-wide waveguide because it was very high (~0.634) and the pump wavelength was almost in its normal dispersion region. As discussed in the previous section, a decrease in *δ* results in an increase in the spectral shift accompanied by a gradual drop in the signal amplitude, explaining the observed continuous decrease in the signal with greater waveguide width. The maximum signal of ~2.3 μm was obtained with 560 nm wide waveguide. Here all spectra are only plotted from 2.1 μm because we used a long-pass filter to avoid any spectrometer (Thorlab FTIR) artifacts. We also observed that for all the waveguides from 500 to 600 nm, no SDWs below the 1st ZDW were generated.

We learn from these results that the LDWs for different waveguides remained lower in power than the signal generated at 2.3 μm in the W1 waveguide (shown in green in Figure 7a). This result can be explained by two effects. (a) The signal from W1 at 2.3 μm is due to the high peak power fundamental soliton. That is because temporally the shortest (high peak power) soliton shifts to the longest possible wavelength after the fission of the higher-order soliton into many fundamental solitons^41^. The signal at 2.3 μm fits the condition for a fundamental soliton (*N*=1) with the peak power of ~2 W and pulse width of <75 fs. (b) The dispersive waves are generally weaker, that is, their power is within 1–5% of the high-power solitons generated after fission, and they also spread out temporally and spectrally over a wide bandwidth, thus limiting the peak and average power in the relevant region (that is, *f*). We also investigated the variation in the *f* signal with varying pump energy (30, 21, and 10 pJ) as shown in Figure 7b and 7c. We observe that both the soliton and LDW are shifted in wavelength due to the power-dependence of the self-phase modulation (SPM). Nevertheless, as the *f* LDW is not at the edge of the spectrum, unlike the *f* soliton, the amplitude of the *f* signal in Figure 7c is not affected as much by variation of power as shown in Figure 7b.

**Figure 8:**
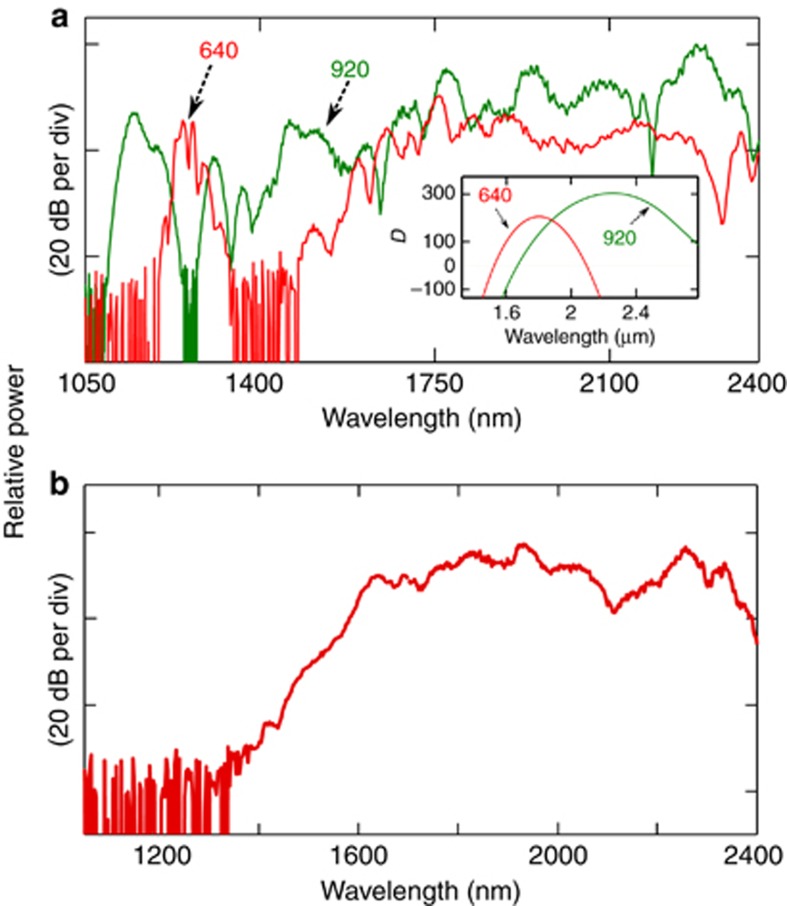
(**a**) Comparison of supercontinuum spectra of waveguide W1 (green) and W3 (red). Inset shows the dispersion of the two waveguides. Unit of D is ps nm^−1^ km^−1^. (**b**) Full SC spectrum of 560-nm-wide waveguide pumped at 1.94 μm with coupled energy of 30 pJ.

These results clearly suggest that even in the upper limit of the LDW generation (that is, pumping only for efficient LDW), the soliton-based signal energy is comparable to, if not significantly higher than, the most efficient LDW signal. Moreover, the pulse width of the LDW is normally several picoseconds. From the simulation we predict it to be >70 times wider than the high peak power fundamental soliton pulse width. This results in the reduction of peak power for LDW for applications requiring further nonlinear conversion. As mentioned, we tested only the upper limit of the LDW so far, however, for octave-spanning applications in which 1*f* and 2*f* signals are generated with the help of a waveguide with two ZDWs, the LDW will be significantly lower in amplitude than the soliton-based *f* signal. To test that, we designed and measured a waveguide with two neighboring ZDWs and compared it with W1, as shown in Figure 8a. Here the two spectra are plotted together; the green curve shows the data taken from Figure 2 and the red curve is for a waveguide W3, with width, height and slab thickness of 640, 280, and 100 nm, respectively. The dispersion curves are shown in the inset. The 1st and 2nd ZDWs are 1.526 and 2.095 μm, respectively, for the W3 waveguide. The pump parameters were the same as for W1 in Figure 2.

As expected, the LDW is significantly weaker than the soliton from W1, ~17 dB weaker around 2.3 μm. This is due to a significant amount of power being required for efficient SDW generation. Moreover, the SDW of the W3 waveguide was not as short in wavelength as the W1 waveguide even though the *δ* was smaller (~0.017 at 1.7 μm) than for W1 (~0.022 at 1.9 μm). This is explained by the fact that the SDW of W3 is generated by a significantly low power soliton located at ~1.7 μm and not directly by the pump pulse because the pump wavelength is in the negative *β*_3_ region, causing most of the pump energy to go into the LDW. Moreover, higher-order dispersion effects become important in W3 (near 1.7 μm), which can reduce the SDW that is aggravated by the higher TPA and lower *n*_2_ at ~1.7 μm. We also observe that the signal in the 1400–1600 nm range is weaker in W3 than in W1. That is due to the *δ* being higher for W1; hence, it has more energy for SDW than in the W3 waveguide, as explained previously (see Equation (3)). In addition, insufficient SPM-based spectral broadening occurs close to the 1st ZDW in the anomalous region of W3.

To generate both efficient SDW and LDW, a careful design consideration for *β*_3_ is required. Simply modifying the waveguide design of W3 to further reduce the dispersion would not help because this would result in higher *δ*, causing a smaller frequency shift. Conversely, by increasing the dispersion to decrease the *δ*, the SPM-based broadening is compromised due to high *β*_2_; hence, not enough energetic signals are generated to radiate into the SDW, as is evidenced in Figure 8b, where we do not observe any SDW. In Figure 8b, we plotted the full spectrum for one of the waveguides described (560 nm) in Figure 7. All other waveguides show similar response below 1st ZDW. In addition, shifting the dispersion such that the 1st ZDW is near the pump wavelength (1.9 μm) would result in the LDW being generated in the regions where the silica substrate loss can be overwhelming (>2.5 um^25^). Nevertheless, a lower *δ* can be obtained over a wide range, for example, by reducing the *β*_3_ over such a range by designing a flatter *β*_2_^54^. The *β*_2_ should be low enough for efficient SPM process to produce a wider and flatter SC for a waveguide with two ZDWs. However, generating LDW that is stronger than the solitons will still be challenging.

## Conclusion

In this work, we demonstrated that a coherent supercontinuum can be generated over the entire near-IR transparency window of silicon. Based on this advance together with the recently demonstrated silicon second-harmonic generator^34^ and a chip-scale thulium mode-locked laser source^35, 55^, various applications can be envisioned, such as CMOS-compatible chip-scale optical metrology, frequency synthesis and precision spectroscopy. Moreover, the strong SC signal around 1550 nm bodes well for frequency synthesis for telecom applications^56^, in which case a silicon SC device can be integrated together with silicon-based erbium-doped Al_2_O_3_ CW lasers^57^. Such a synthesizer would leverage established integrated germanium detectors that are not available in the mid-IR^58^. Moreover, we show experimentally that to generate efficient DW, optimization of the third-order dispersion should be considered for a nonlinear waveguide design along with the design for nonlinear parameter *γ*^59^. We also demonstrate the advantage of soliton-based *f* signal over LDW for obtaining high peak power at *f* for applications in optical frequency metrology. Such a silicon-based soliton has an additional advantage of being coherent due to the lack of a broad Raman gain in silicon compared to photonics crystal fiber, where a significant amount of soliton spectral shift is induced by Raman effects^60^.

There is still room for improvement. For example, a flatter SC can be achieved with optimally designed tapered waveguides that are also promising for relaxing the requirement of sub 100 fs driver pulses for coherent SCG^61, 62, 63, 64^. Further improvements in *f* and *2f* spectra can be obtained by implementing Bragg-gratings in the SC waveguides for selective enhancement of the signal^65^. Furthermore, mode-locked lasers based on holmium can be used for pumping SC because silicon shows the highest *n*_2_ ~2.1 μm^66^. If combined with high-repetition-rate lasers, this can lead to an increased signal strength over the entire SC spectrum.

Together with other relevant demonstrations of silicon-based SCG^23, 26^, this work establishes silicon as an important platform for supercontinuum generation and nonlinear photonics in general.

## References

[bib1] Alfano RR, Shapiro SL. Observation of self-phase modulation and small-scale filaments in crystals and glasses. Phys Rev Lett 1970; 24: 592–594.

[bib2] Ranka JK, Windeler RS, Stenza AJ. Visible continuum generation in air-silica microstructure optical fibers with anomalous dispersion at 800 nm. Opt Lett 2000; 25: 25–27. 18059770 10.1364/ol.25.000025

[bib3] Udem T, Holzwarth R, Hänsch TW. Optical frequency metrology. Nature 2002; 416: 233–237. 11894107 10.1038/416233a

[bib4] Kano H, Hamaguchi H. Characterization of a supercontinuum generated from a photonic crystal fiber and its application to coherent Raman spectroscopy. Opt Lett 2003; 28: 2360–2362. 14680182 10.1364/ol.28.002360

[bib5] Hartl L, Li XD, Chudoba C, Ghanta RK, Ko TH et al. Ultrahigh-resolution optical coherence tomography using continuum generation in an air-silica microstructure optical fiber. Opt Lett 2001; 26: 608–610. 18040398 10.1364/ol.26.000608

[bib6] Morioka T, Okamoto K, Ishii M, Saruwatari M. Low-noise, pulsewidth tunable picosecond to femtosecond pulse generation by spectral filtering of wideband supercontinuum with variable bandwidth arrayed-waveguide grating filters. Electron Lett 1996; 32: 836–837.

[bib7] Morioka T, Mori K, Saruwatari M. More than 100-wavelength-channel picosecond optical pulse generation from single laser source using supercontinuum in optical fibres. Electron Lett 1993; 29: 862–864.

[bib8] Jalali B, Fathpour S. Silicon photonics. J Lightwave Technol 2006; 24: 4600–4615.

[bib9] Soref R. The past, present, and future of silicon photonics. IEEE J Sel Topics Quantum Electron 2006; 12: 1678–1687.

[bib10] Zhang L, Agarwal AM, Kimerling LC, Michel J. Nonlinear group IV photonics based on silicon and germanium: from near-infrared to mid-infrared. Nanophotonics 2014; 3: 247–268.

[bib11] Leuthold J, Koos C, Freude W. Nonlinear silicon photonics. Nat Photonics 2010; 4: 535–544.

[bib12] Yin LH, Agrawal GP. Impact of two-photon absorption on self-phase modulation in silicon waveguides. Opt Lett 2007; 32: 2031–2033. 17632633 10.1364/ol.32.002031

[bib13] Yin LH, Qiang L, Agrawal GP. Soliton fission and supercontinuum generation in silicon waveguides. Opt Lett 2007; 32: 391–393. 17356663 10.1364/ol.32.000391

[bib14] Leo F, Gorza SP, Safioui J, Kockaert P, Coen S et al. Dispersive wave emission and supercontinuum generation in a silicon wire waveguide pumped around the 1550 nm telecommunication wavelength. Opt Lett 2014; 39: 3623–3626. 24978552 10.1364/OL.39.003623

[bib15] Halir R, Okawachi Y, Levy JS, Foster MA, Lipson M et al. Ultrabroadband supercontinuum generation in a CMOS-compatible platform. Opt Lett 2012; 37: 1685–1687. 22627537 10.1364/OL.37.001685

[bib16] Moss DJ, Morandotti R, Gaeta AL, Lipson M. New CMOS-compatible platforms based on silicon nitride and Hydex for nonlinear optics. Nat Photonics 2013; 7: 597–607.

[bib17] Yu Y, Gai X, Ma P, Choi DY, Yang ZY et al. A broadband, quasi-continuous, mid-infrared supercontinuum generated in a chalcogenide glass waveguide. Laser Photonics Rev 2014; 8: 792–798.

[bib18] Ettabib MA, Xu L, Bogris A, Kapsalis A, Belal M et al. Broadband telecom to mid-infrared supercontinuum generation in a dispersion-engineered silicon germanium waveguide. Opt Lett 2015; 40: 4118–4121. 26368726 10.1364/OL.40.004118

[bib19] Dave UD, Ciret C, Gorza SP, Combrie S, De Rossi A et al. Dispersive-wave-based octave-spanning supercontinuum generation in InGaP membrane waveguides on a silicon substrate. Opt Lett 2015; 40: 3584–3587. 26258363 10.1364/OL.40.003584

[bib20] Hickstein DD, Jung H, Carlson DR, Lind A, Coddington I et al. Ultrabroadband supercontinuum generation and frequency-comb stabilization using on-chip waveguides with both cubic and quadratic nonlinearities. Phys Rev Appl 2017; 8: 014025.

[bib21] Safioui J, Leo F, Kuyken B, Gorza SP, Selvaraja SK et al. Supercontinuum generation in hydrogenated amorphous silicon waveguides at telecommunication wavelengths. Opt Express 2014; 22: 3089–3097. 24663599 10.1364/OE.22.003089

[bib22] Shen L, Healy N, Xu L, Cheng HY, Day TD et al. Four-wave mixing and octave-spanning supercontinuum generation in a small core hydrogenated amorphous silicon fiber pumped in the mid-infrared. Opt Lett 2014; 39: 5721–5724. 25360968 10.1364/OL.39.005721

[bib23] Kuyken B, Ideguchi T, Holzner S, Yan M, Hänsch TW et al. An octave-spanning mid-infrared frequency comb generated in a silicon nanophotonic wire waveguide. Nat Commun 2015; 6: 6310. 25697764 10.1038/ncomms7310 PMC4346629

[bib24] Kuyken B, Liu XP, Osgood RM, Baets R, Roelkens G et al. Mid-infrared to telecom-band supercontinuum generation in highly nonlinear silicon-on-insulator wire waveguides. Opt Express 2011; 19: 20172–20181. 21997028 10.1364/OE.19.020172

[bib25] Lau RKW, Lamont MRE, Griffith AG, Okawachi Y, Lipson M et al. Octave-spanning mid-infrared supercontinuum generation in silicon nanowaveguides. Opt Lett 2014; 39: 4518–4521. 25078217 10.1364/OL.39.004518

[bib26] Singh N, Hudson DD, Yu Y, Grillet C, Jackson SD et al. Midinfrared supercontinuum generation from 2 to 6 μm in a silicon nanowire. Optica 2015; 2: 797–802.

[bib27] Nader N, Maser DL, Cruz FC, Fredrick C, Ycas G et al Coherent On-Chip Spectral-Engineered Mid-IR Frequency Comb Generation in Si Waveguides. Proceedings of 2017 Conference on Lasers and Electro-Optics; 14–19 May 2017; San Jose, CA, USA. Optical Society of America: San Jose, CA, USA, 2017.

[bib28] Baehr-Jones T, Spott A, Ilic R, Spott A, Penkov B et al. Silicon-on-sapphire integrated waveguides for the mid-infrared. Opt Express 2010; 18: 12127–12135. 20588335 10.1364/OE.18.012127

[bib29] Singh N, Casas-Bedoya A, Hudson DD, Read A, Mägi E et al. Mid-IR absorption sensing of heavy water using a silicon-on-sapphire waveguide. Opt Lett 2016; 41: 5776–5779. 27973499 10.1364/OL.41.005776

[bib30] Singh N, Hudson DD, Eggleton BJ. Silicon-on-sapphire pillar waveguides for Mid-IR supercontinuum generation. Opt Express 2015; 23: 17345–17354. 26191744 10.1364/OE.23.017345

[bib31] Chiles J, Fathpour S. Single-mode and single-polarization photonics with anchored-membrane waveguides. Opt Express 2016; 24: 19337–19343. 27557212 10.1364/OE.24.019337

[bib32] Zhang L, Lin Q, Yue Y, Yan Y, Beausoleil RG et al. Silicon waveguide with four zero dispersion wavelengths and its application in on-chip octave-spanning supercontinuum generation. Opt Express 2012; 20: 1685–1690. 22274510 10.1364/OE.20.001685

[bib33] Leo F, Gorza SP, Coen S, Kuyken B, Roelkens G. Coherent supercontinuum generation in a silicon photonic wire in the telecommunication wavelength range. Opt Lett 2015; 40: 123–126. 25531625 10.1364/OL.40.000123

[bib34] Timurdogan E, Poulton CV, Byrd MJ, Watts MR. Electric field-induced second-order nonlinear optical effects in silicon waveguides. Nat Photonics 2017; 11: 200–206.

[bib35] Callahan PT, Shtyrkova K, Li NX, Magden ES, Purnawirman P et al Fully-Integrated CMOS-Compatible Q-Switched Laser at 1.9 μm Using Thulium-Doped Al2O3. Proceedings of 2017 Conference on Lasers and Electro-Optics; 14–19 May 2017; San Jose, CA, USA. Optical Society of America: San Jose, CA, USA, 2017.

[bib36] Bristow AD, Rotenberg N, van Driel HM. Two-photon absorption and Kerr coefficients of silicon for 850-2200 nm. Appl Phys Lett 2007; 90: 191104.

[bib37] Poletti F, Horak P. Dynamics of femtosecond supercontinuum generation in multimode fibers. Opt Express 2009; 17: 6134–6147. 19365436 10.1364/oe.17.006134

[bib38] Almeida VR, Panepucci RR, Lipson M. Nanotaper for compact mode conversion. Opt Lett 2003; 28: 1302–1304. 12906070 10.1364/ol.28.001302

[bib39] Corcoran B, Monat C, Grillet C, Moss DJ, Eggleton BJ et al. Green light emission in silicon through slow-light enhanced third-harmonics generation in photonics-crystal waveguides. Nat Photonics 2009; 3: 206–210.

[bib40] Dudley JM, Coen S. Coherence properties of supercontinuum spectra generated in photonic crystal and tapered optical fibers. Opt Lett 2002; 27: 1180–1182. 18026400 10.1364/ol.27.001180

[bib41] Agrawal GNonlinear Fiber Optics, 5th edn. Amsterdam: Elsevier; 2012.

[bib42] Bertolotti M, Ferrari A, Sereda L. Coherence properties of nonstationary polychromatic light source*s*. J Opt Soc Am B 1995; 12: 341–347.

[bib43] Bellini M, Hänsch TW. Phase-locked white-light continuum pulses: toward a universal optical frequency-comb synthesizer. Opt Lett 2000; 25: 1049–1051. 18064269 10.1364/ol.25.001049

[bib44] Gu X, Kimmel M, Shreenath AP, Trebino R, Dudley JM et al. Experimental studies of the coherence of microstructure-fiber supercontinuum. Opt Express 2003; 11: 2697–2703. 19471384 10.1364/oe.11.002697

[bib45] Lu F, Knox WH. Generation of a broadband continuum with high spectral coherence in tapered single-mode optical fibers. Opt Express 2004; 12: 347–353. 19471544 10.1364/opex.12.000347

[bib46] Herrmann J, Griebner U, Zhavoronkov N, Husakou A, Nickel D et al. Experimental evidence for supercontinuum generation by fission of higher-order solitons in photonic fibers. Phys Rev Lett 2001; 88: 173901. 10.1103/PhysRevLett.88.173901 12005754

[bib47] Akhmediev N, Karlsson M. Cherenkov radiation emitted by solitons in optical fibers. Phys Rev A 1995; 41: 2602–2607. 10.1103/physreva.51.2602 9911876

[bib48] Roy S, Bhadra SK, Agrawal GP. Dispersive waves emitted by solitons perturbed by third-order dispersion inside optical fibers. Phys Rev A 2009; 79: 023824. 10.1364/ol.34.002072 19572003

[bib49] Roy S, Bhadra SK, Agrawal GP. Effects of higher-order dispersion on resonant dispersive waves emitted by solitons. Opt Lett 2009; 34: 2072–2074. 19572003 10.1364/ol.34.002072

[bib50] Paschotta R. Noise of mode-locked lasers (part1): numerical model. Appl Phys B 2004; 79: 153–162.

[bib51] Genty G, Lehtonen M, Ludvigsen H, Kaivola M. Enhanced bandwidth of supercontinuum generated in microstructured fibers. Opt Express 2004; 12: 3471–3480. 19483874 10.1364/opex.12.003471

[bib52] Frosz MH, Falk P, Bang O. The role of the second zero-dispersion wavelength in generation of supercontinua and bright-bright soliton-pairs across the zero-dispersion wavelength. Opt Express 2005; 13: 6181–6192. 19498630 10.1364/opex.13.006181

[bib53] Hilligsøe KM, Andersen TV, Paulsen HN, Nielsen CK, Mølmer K et al. Supercontinuum generation in a photonic crystal fiber with two zero dispersion wavelengths. Opt Express 2004; 12: 1045–1054. 19474920 10.1364/opex.12.001045

[bib54] Kerrinckx E, Bigot L, Douay M, Quiquempois Y. Photonic crystal fiber design by means of a genetic algorithm. Opt Express 2004; 12: 1990–1995. 19475033 10.1364/opex.12.001990

[bib55] Li NX, Purnawirman P, Su Z, Magden ES, Callahan PT et al. High-power thulium lasers on a silicon photonics platform. Opt Lett 2017; 42: 1181–1184. 28295078 10.1364/OL.42.001181

[bib56] Spencer DT, Bluestone A, Bowers JE, Briles TC, Diddams SA et al Towards an Integrated-Photonics Optical-Frequency Synthesizer with <1 Hz Residual Frequency Noise. Proceedings of 2017 Optical Fiber Communication Conference and Exposition; 19 March 2017; Los Angeles, CA, USA. OFC: Los Angeles, CA, USA, 2017.

[bib57] Singh G, Purnawirman, Bradley JDB, Li N, Magden ES et al. Resonant pumped erbium-doped waveguide lasers using distributed Bragg reflector cavities. Opt Lett 2016; 41: 1189–1192. 26977666 10.1364/OL.41.001189

[bib58] Michel J, Liu JF, Kimerling LC. High-performance Ge-on-Si photodetectors. Nat Photonics 2010; 4: 527–534.

[bib59] Foster MA, Moll KD, Gaeta AL. Optimal waveguide dimensions for nonlinear interactions. Opt Express 2004; 12: 2880–2887. 19483803 10.1364/opex.12.002880

[bib60] Klenner A, Mayer AS, Johnson AR, Luke K, Lamont MRE et al. Gigahertz frequency comb offset stabilization based on supercontinuum generation in silicon nitride waveguides. Opt Express 2016; 24: 11043–11053. 27409927 10.1364/OE.24.011043

[bib61] Mori K, Takara H, Kawanishi S. Analysis and design of supercontinuum pulse generation in a single-mode optical fiber. J Opt Soc Am B 2001; 18: 1780–1792.

[bib62] Genty G, Coen S, Lacourt PA, Dudley JM Highly Coherent Supercontinuum Generation in Dispersion Increasing Fibers. Proceedings of 2017 Nonlinear Photonics; 2 September 2007; Quebec City, Canada. Optical Society of America: Quebec City, Canada, 2007.

[bib63] Zhang HN, Li P. Ultra-flat supercontinuum generation in cascaded photonic crystal fiber with picosecond fiber laser pumping. Opt Commun 2016; 372: 60–63.

[bib64] Ciret C, Gorza SP. Generation of ultra-broadband coherent supercontinua in tapered and dispersion-managed silicon nanophotonic waveguides. J Opt Soc Am B 2017; 34: 1156–1162.

[bib65] Westbrook PS, Nicholson JW, Feder KS, Li Y, Brown T. Supercontinuum generation in a fiber grating. Appl Phys Lett 2004; 85: 4600–4602.

[bib66] Wang T, Venkatram N, Gosciniak J, Cui Y, Qian G et al. Multi-photon absorption and third-order nonlinearity in silicon at mid-infrared wavelengths. Opt Express 2013; 21: 32192–32198. 24514813 10.1364/OE.21.032192

